# Internet-Delivered Cognitive Behavioral Therapy for Generalized Anxiety Disorder in Nationwide Routine Care: Effectiveness Study

**DOI:** 10.2196/29384

**Published:** 2022-03-24

**Authors:** Ville Ritola, Jari Olavi Lipsanen, Satu Pihlaja, Eero-Matti Gummerus, Jan-Henry Stenberg, Suoma Saarni, Grigori Joffe

**Affiliations:** 1 Department of Psychiatry Helsinki University Hospital and University of Helsinki Helsinki Finland; 2 Department of Psychology and Logopedics University of Helsinki Helsinki Finland

**Keywords:** CBT, iCBT, cognitive behavioral therapy, routine care, generalized anxiety disorder, internet, web-based, digital health, mental health

## Abstract

**Background:**

Therapist-supported, internet-delivered cognitive behavioral therapy (iCBT) is efficacious for generalized anxiety disorder (GAD), but few studies are yet to report its effectiveness in routine care.

**Objective:**

In this study, we aim to examine whether a new 12-session iCBT program for GAD is effective in nationwide routine care.

**Methods:**

We administered a specialized, clinic-delivered, therapist-supported iCBT for GAD in 1099 physician-referred patients. The program was free of charge for patients, and the completion time was not predetermined. We measured symptoms with web-based questionnaires. The primary measure of anxiety was the GAD 7-item scale (GAD-7); secondary measures were, for pathological worry, the Penn State Worry Questionnaire and, for anxiety and impairment, the Overall Anxiety Severity and Impairment Scale.

**Results:**

Patients completed a mean 7.8 (SD 4.2; 65.1%) of 12 sessions, and 44.1% (485/1099) of patients completed all sessions. The effect size in the whole sample for GAD-7 was large (Cohen *d*=0.97, 95% CI 0.88-1.06). For completers, effect sizes were very large (Cohen *d*=1.34, 95% CI 1.25-1.53 for GAD-7; Cohen *d*=1.14, 95% CI 1.00-1.27 for Penn State Worry Questionnaire; and Cohen *d*=1.23, 95% CI 1.09-1.37 for Overall Anxiety Severity and Impairment Scale). Noncompleters also benefited from the treatment. Greater symptomatic GAD-7–measured relief was associated with more completed sessions, older age, and being referred from private or occupational care. Of the 894 patients with a baseline GAD-7 score ≥10, approximately 421 (47.1%) achieved reliable recovery.

**Conclusions:**

This nationwide, free-of-charge, therapist-supported HUS Helsinki University Hospital–iCBT for GAD was effective in routine care, but further research must establish effectiveness against other treatments and optimize the design of iCBT for GAD for different patient groups and individual patients.

## Introduction

### Background

Generalized anxiety disorder (GAD) [1] is a common but underdiagnosed and undertreated condition [2,3]. The global annual prevalence of GAD in high-income countries may be 2.3%, and this disorder is associated with increased functional impairment [4].

Pharmacotherapy and psychotherapy are considered first-line GAD treatments [5,6]. Among psychotherapies, cognitive behavioral therapy (CBT) is the most studied, with large effect sizes (Hedges *g*=0.90 [7]). However, traditional face-to-face CBT is relatively resource-consuming and limited in access.

The need to resolve the accessibility and affordability challenges of face-to-face CBT led to the development of internet-delivered CBT (iCBT), with notable work occurring in Sweden, the Netherlands, Denmark, Australia, and Canada, for example [8]. A typical iCBT intervention is location-independent and includes a program with therapeutic content and homework. It is accessible 24/7 through a web-based platform or mobile app, with or without therapist support. In therapist-supported iCBT, remote therapist support is most often asynchronous. While the intensity of therapist support in iCBT can vary, it requires far less therapist time compared with face-to-face or real-time remote psychotherapy and thus may be more cost-effective [9,10].

As demonstrated in recent meta-analyses of randomized controlled trials (RCTs) [9,11-16], therapist-supported iCBTs are efficacious in several psychiatric disorders and may be as efficacious as short-term face-to-face CBT. Therapist-supported iCBTs for depression and anxiety have higher efficacy and adherence than unguided programs do [12,17-19], which is likely due to the high level of client–therapist alliance, comparable with that in face-to-face therapies [20]. In general, iCBTs reduce symptomatic deterioration more than waiting lists (3.1% or 5.8% vs 17.4% [13,21]). For therapist-supported iCBT for GAD, efficacy has also been established for both diagnosis-specific and transdiagnostic programs [9,16].

However, RCT efficacy studies, being the gold standard for clinical evidence, have their own shortcomings. Participants are more likely to be highly motivated, have had thorough screening, and have received more intensive treatment than in routine care. Therefore, RCT-validated treatments may or may not yield similar results or level of adherence in routine care depending on whether the criteria are lax [22-24]. Addressing these limitations, routine care studies are now accepted as valid scientific evidence for their clinical effectiveness and safety [25]. Although there is strong real-world evidence for the effectiveness of therapist-supported iCBT in general [8,9,13], studies focusing on GAD are limited.

We identified 5 publications comprising 7 therapist-supported iCBT interventions focusing on GAD in routine care using 3 different programs at 2 clinics. ThisWayUp clinic in Australia performed 6 interventions comprising 2 programs [23,26-28], and Online Therapy Unit in Canada performed 1 intervention [29]. These studies mainly reported large intention-to-treat (ITT) effect sizes (0.91-1.30), although 1 outlier study reported effects as large as 2.06 and 2.10 [28]. ThisWayUp trials reported full completion rates of 36% to 55%. Although these 3 programs demonstrated the overall feasibility of iCBT for GAD, the recruitment schemes, program length, and therapist support intensity in these trials varied widely (see the Design Comparison section in the Discussion section), and the Online Therapy Unit’s trial was relatively small. More routine care studies are needed to confirm their conclusions and elucidate the optimal program design.

The HUS Helsinki University Hospital has developed and is providing nationwide, original Finnish language therapist-supported iCBT programs for several psychiatric conditions (further referred to as HUS-iCBTs), including one for GAD. Intake for the 12-session program requires a physician’s referral, it is free of charge, and therapist support is provided centrally by a specialized clinic. As the combination of setting and design of the HUS-iCBT for GAD differs from those studied earlier, the effectiveness of these programs may differ.

### Objective

The aim of this study is to examine the effectiveness of HUS-iCBT for GAD in routine care. We hypothesized that the intervention would have a large overall effect size and the patients’ completion rate would be comparable (36%-55%) with the reported routine care studies.

## Methods

### Setting and Design

The HUS-iCBTs were delivered centrally by the iCBT clinic at HUS Psychiatry. The interventions were free of charge, diagnosis-specific, and therapist-supported programs. All physicians in Finland can refer their patients to therapy. The referring physicians received support from the instructions on the web. A specially trained mental health professional screened all referrals centrally. We deliberately implemented these inclusion procedures to enable virtually unlimited nationwide access for the clinical population despite the limited number of therapists.

The study was an observational, nationwide, open-label, real-world trial.

### Participants

Participants were recruited from those entering HUS-iCBT for GAD between February 2016 and December 2018. To be accepted for the treatment, patients had to (1) be diagnosed with GAD (ICD-10 [1]), (2) have an email address, and (3) have an internet-based bank account or mobile ID (the means of official e-identification in Finland). Comorbidity (other than the exclusion criteria) and concomitant pharmacological and psychological treatments were allowed, as the intervention was part of routine care. Exclusion criteria were acute psychosis or mania, severe personality disorders, severe suicidality, or neurological or neuropsychiatric disorder with cognitive decline. These were screened centrally from the physician’s referral before the referral was accepted. The only additional inclusion criterion for the study was a baseline score of ≥8 on the GAD 7-item scale (GAD-7 [30]).

### Ethical Considerations

The patients provided informed consent after the first log-in. The study protocol was approved by the ethics committee of the HUS and pertinent institutional authorities.

### Intervention and Procedure

The new iCBT program for GAD was designed by an expert group to be diagnosis-specific because the existing evidence base was clearly the strongest at the time, in 2013. This new program consisted of 12 consecutive sessions and a follow-up session 3 months after treatment completion. The program was theoretically based on several models of GAD and anxiety, including aspects of the cognitive avoidance model [31], model of intolerance of uncertainty [32], metacognitive therapy [33], and Acceptance and Commitment Therapy [34], as well as social aspects, such as assertiveness training. The sessions included text and videos, as well as educational illustrations, example stories, therapeutic exercises, and homework (Textbox 1).

After approval, the patients received an email and a letter prompting them to sign in and begin treatment. A schedule of 1 session per week was recommended, and a minimum 24-hour waiting period was enforced between sessions to encourage daily life practice. The program sent email prompts for arriving messages and, after 2 weeks of inactivity, log-in reminders. Nevertheless, no maximum completion time was required if the patient remained active in therapy.

Although HUS-iCBT for GAD was therapist-supported, several persuasive elements used in unguided iCBT programs [35] were originally included and are listed in Textbox 2. In total, 7 elements were used (or 6, if discounting tunneling as the original authors did).

**Content by session.**

**Session titles**

- Session 1: Introduction to HUS Helsinki University Hospital–internet-delivered cognitive behavioral therapy program, cognitive behavioral therapy model, and generalized anxiety disorder
- Session 2: Bodily stress response, worry and relaxation
- Session 3: Worry and avoidance, intolerance of uncertainty
- Session 4: Experiential Avoidance, Core Beliefs
- Session 5: Negative beliefs about worrying, challenging worrying
- Session 6: Positive beliefs about worrying, challenging beliefs about worrying
- Session 7: Challenging worrying
- Session 8: Acceptance of worries, acceptance vs submission
- Session 9: Intolerance of uncertainty and reaching for perfection and certainty
- Session 10: Problem solving vs worrying, solvable vs unsolvable worries
- Session 11: Social skills, needs in relationships, assertiveness
- Session 12: Summarizing, warning signs, plan for the future, feedback

**Identified persuasive design principles.**

**Principles and brief example**

- Reduction: simple stepped instructions; for example, worry diary or relaxation
- Tunneling: logical thematic progression
- Self-monitoring: symptom graphs
- Simulation: example stories with avatars
- Rehearsal: web-based worry diary and relaxation training
- Reminders: log-in reminders
- Normative influence: normalization of common generalized anxiety disorder features

### Therapist Support

The therapists in the program were clinical psychologists, psychology students, or nurses with additional therapeutic training, all working at HUS Psychiatry. Each therapist received 1 day of training on internet delivery of CBT, text-based communication, the intervention protocol, and Good Clinical Practice. Therapists received regular group supervision and sought consultation with a senior psychotherapist at any time.

To support patients’ progress, the therapists provided empathic asynchronous feedback with written messages 4 times or more often (if the patient requested) during the therapy. If the patient was inactive for 2 weeks, the therapist pursued contact through an SMS text message or iCBT program. If no reply arrived within a week, further contact was attempted by phone and thereafter by a letter including a 2-week deadline for continuing, after which access to therapy was discontinued.

A web-based report of live data on individual therapists was created during the study to allow for supervision of compliance with the intervention protocol and to prompt support to those failing to ask for support. The report included data on the date of the last log-in and the number of patients not contacted or logged in within the last 2 weeks. The therapist time per patient was not subject to monitoring, but each therapist treated a minimum quota of patients per dedicated working hour. On average, this quota would mean 9 to 11 minutes of working time per patient per week.

### Measures

#### Overview

Symptoms were measured using web-based questionnaires. The primary measure of anxiety was the GAD-7; secondary measures were, for pathological worry, the Penn State Worry Questionnaire (PSWQ) and, for anxiety and impairment, the Overall Anxiety Severity and Impairment Scale (OASIS).

Patients completed the GAD-7 at the beginning of each of the first 11 sessions and at the end of the final 12th session. The 2 secondary measures were filled at the beginning and end of treatment. Each participant received an invitation for a follow-up measurement 3 months after completing the treatment.

#### GAD 7-Item Scale

The GAD-7 is a short, 7-item self-report questionnaire developed to measure GAD diagnostic symptom criteria of the Diagnostic and Statistical Manual of Mental Disorders, Fourth Edition [30]. Its internal consistency (Cronbach α) has generally ranged between .88 in the clinical population and .92 in the general population [36,37]. The test–retest reliability in a study was 0.83 [30]. In this study, Cronbach α was .725 before the treatment and .905 after the treatment.

#### Penn State Worry Questionnaire

The PSWQ is a 16-item questionnaire designed to measure pathological worry, a signature feature of GAD [38]. Internal consistency has ranged from .86 to .95 among various samples [38-40]. PSWQ sensitivity to change is good but less salient than that of GAD-7 [41]. Test–retest reliability has been inconsistent (0.67-0.93) [39,42], so a midrange score of 0.80 was our choice. In our study, the Cronbach α was .865 before the treatment and .931 after the treatment.

#### Overall Anxiety Severity and Impairment Scale

The OASIS is a 5-item scale developed as a brief transdiagnostic measure of anxiety severity and impairment [43]. Internal consistency has ranged from .84 to .93 in 3 large clinical samples [44-46]. OASIS’s sensitivity to change has been established, but its diagnostic accuracy is low [45,47]. Test–retest reliability has been 0.73 in undergraduate samples and 0.82 in clinical samples [43,45] with a midrange value of 0.78 used. Cronbach α in this study was .736 before the treatment and .888 after the treatment.

#### Adherence

We measured the number of completed sessions and the time of therapy, defined as the time between the first and last GAD-7 in-therapy measurements. Only patients who completed all 12 sessions were categorized as completers. The program introduced the main theoretical framework in the first 4 sessions, and the later sessions focused mostly on further implementation of basic principles or introduced secondary content. Hence, we subcategorized noncompleters into two groups a priori: early dropouts (those discontinued before the completion of the fourth session) and late dropouts.

#### Patient Characteristics

We collected patient demographics such as age, registered sex, and municipality class. The municipality class was either urban or nonurban, according to the official Finnish classification [48].

### Statistical Analysis

Our data were a convenience sample of all consented patients who entered treatment after February 2016. Our desired power was 80% and type I error was 0.05 for the main tests and 0.01 for post hoc tests. When comparing 2 independent means, a requirement to detect a small effect size, a minimum of 0.2, would be achieved with a sample of 435 for main tests and 647 for post hoc tests, assuming a 45%/55% completer/noncompleter distribution. For mixed model parameters, we set significance at a conservative *P*<.01, while ensuring that Akaike and Bayesian Information Criteria [49,50] do not indicate worsening fit. The final mixed model was built in a backward stepwise manner.

The primary outcome analysis involved a linear mixed random model that allowed growth modeling to account for the changing pace of recovery at different time points during therapy, the use of all available data, and different intercept estimations for each individual. GAD-7 served as the dependent variable, and we modeled a growth curve using linear, quadratic, and cubic terms of the GAD-7 observation time. To control for the effect of *dose* of and time on therapy, we also tried a full model with the main effects and interactions of the GAD-7 observation session. We also input the main effects of gender, age, referral source, municipality class, and completion status, along with their linear combinations with time.

The ITT and noncompleter groups’ effect sizes for GAD-7 were estimated with mixed model–estimated marginal means. This approach generally performs better than a pure last observation carried forward (LOCF) at handling dropout bias, especially in larger samples (n>400 [51]). The method used to calculate effect sizes was that of Morris and DeShon [52] (equations 13 and 12). Calculations also used the baseline SD and observed pre–post correlation.

We calculated clinical change indexes using the LOCF values. Reliable change was defined as Reliable Change Index (RCI [53]), and recovery was defined as transitioning below the clinical cutoff on the primary outcome measure, GAD-7. The RCI and clinical cutoff together provide stringent criteria for both response and recovery [54]. Test–retest reliability obtained from earlier normative studies allowed the calculation of the RCI. We explored recovery for those with a baseline GAD-7 score of ≥10 to avoid confounding recovery rates with those of patients who had already fulfilled the criterion at baseline. In reliable recovery, the patient fulfilled the criteria for both clinical recovery and RCI. Full symptomatic recovery (remission) was defined as a posttreatment score <5 on the GAD-7.

We used SPSS Statistics 22 (IBM Corporation) for the analyses [55].

## Results

### Patient Flow, Baseline Characteristics, and Adherence

**Figure 1:**
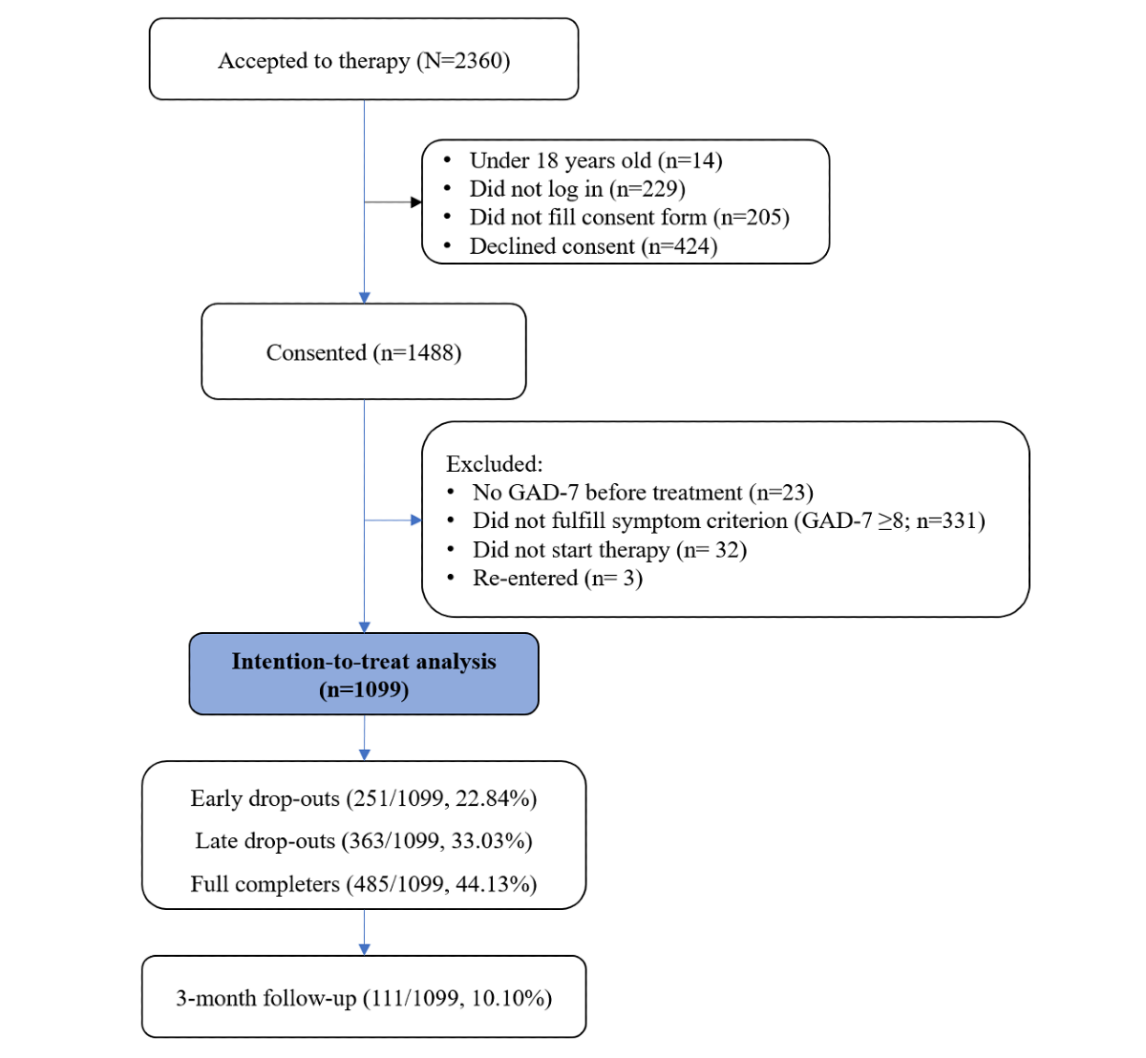
Patient flowchart. GAD-7: Generalized Anxiety Disorder 7-item scale.

Of all referrals to the clinic, those rejected during the recruitment period amounted to only 0.6%. Of the 1912 patients who completed the consent form, 1488 (77.82%) provided their consent (Figure 1).

Of the 1099 patients analyzed, 485 (44.13%) fully completed the HUS-iCBT, and a further 363 (33.03%) completed at least the first 4 sessions, meaning that 251 (22.84%) patients dropped out early. Those who completed from 4 to 11 sessions were considered as late dropouts. On average, patients completed 7.8 (SD 4.2; 65.1%) of the 12 sessions. The average time on therapy (time between pretreatment and last measurements) in the whole sample was 128 (SD 97) days, 171 (SD 95) days for completers, 36 (SD 44) days for early dropouts, and 131 (SD 84) days for late dropouts.

At baseline, completers and noncompleters differed in their proportions regarding referral source, municipality class, average age, and OASIS scores (Table 1). The average age of completers was 4.9 years higher with their average OASIS score being 0.7 points higher than the score of noncompleters. Statistically significant differences did not emerge in gender distribution or in the average GAD-7 and PSWQ scores.

In post hoc analyses, completion was more likely among patients referred from private or occupational care (50.4%) than among those from primary care (39.1%; *χ*^2^_1_=9.675; *P*=.004). Baseline OASIS scores differed significantly between early dropouts and both late dropouts (t_612_=4.297; *P*<.001) and completers (t_734_=6.041; *P*<.001), but not between late dropouts and completers (t_846_=1.625; *P=*.105). The average baseline OASIS scores were 13.3 for early dropouts, 12.3 for late dropouts, and 12.0 for completers.

In total, 30 therapists treated an average of 37 (SD 38) study patients. Of the 1099 patients, 1097 (99.81%) received at least one message from their therapist, and 796 (72.43%) sent one or more messages to their therapist. Completers sent a message more often (420/485, 86.6% patients) than noncompleters (376/614, 61.2% patients). Therapists sent, on average, 8.4 (SD 4.7) messages and patients (those who did) sent 4.0 (SD 4.2) messages. Of those patients who did send messages, the average number of messages that completers sent (4.9, SD 4.8) was greater than that of noncompleters (3.1, SD 3.1). Therapists sent, on average, more messages to completers (11.6, SD 4.4) than to noncompleters (5.9, SD 3.1).

**Table 1:** Baseline characteristics.

Characteristics			Total sample (N=1099)		Completers (n=485)		Noncompleters (n=614)		Completers vs noncompleters				
									Test statistic			*P* value	
**Gender, n (%)**										*χ*^2^_1_=2.240			.13
	Female	849 (77.3)		385 (79.4)		464 (75.6)							
	Male	250 (22.7)		100 (20.6)		150 (24.4)							
**Referral source, n (%)**										*χ*^2^_4_=16.872			.002
	Primary care	601 (54.7)		235 (48.5)		366 (59.6)							
	Private or occupational	339 (30.8)		171 (35.3)		168 (27.4)							
	Student health care	50 (4.5)		24 (4.9)		26 (4.2)							
	Psychiatry	55 (5)		25 (5.2)		30 (4.9)							
	Unspecified	54 (4.9)		33 (6.8)		21 (3.4)							
**Municipality class, n (%)**										*χ*^2^_1_=8.469			.004
	Urban	966 (87.9)		412 (84.9)		554 (90.2)							
	Nonurban	130 (11.8)		73 (7.2)		57 (5.7)							
Age (years), mean (SD)			33.3 (12.2)		36.0 (13.1)		31.1 (12.2)		t_935_=6.578			<.001	
GAD-7^a^, mean (SD)			13.2 (3.6)		13.1 (3.6)		13.3 (3.6)		t_1038_=0.941			.35	
PSWQ^b^, mean (SD)			64.4 (9.8)		64.7 (9.9)		64.2 (9.7)		t_1031_=0.723			.47	
OASIS^c^, mean (SD)			12.4 (2.8)		12.0 (2.8)		12.7 (2.8)		t_1046_=4.216			<.001	

^a^GAD-7: Generalized Anxiety Disorder 7-item scale.

^b^PSWQ: Penn State Worry Questionnaire.

^c^OASIS: Overall Anxiety Severity and Impairment Scale.

### Primary Outcome Model

The building of the primary mixed model is described in Multimedia Appendix 1 and Textbox 1. The model was built backward stepwise, with all terms of interest entered into the model. Eliminated terms were gender, both in average effects and time interaction; municipality class, both in average effects and time interaction; age in average effects; and completion status, both in average effects and session interaction.

**Figure 2:**
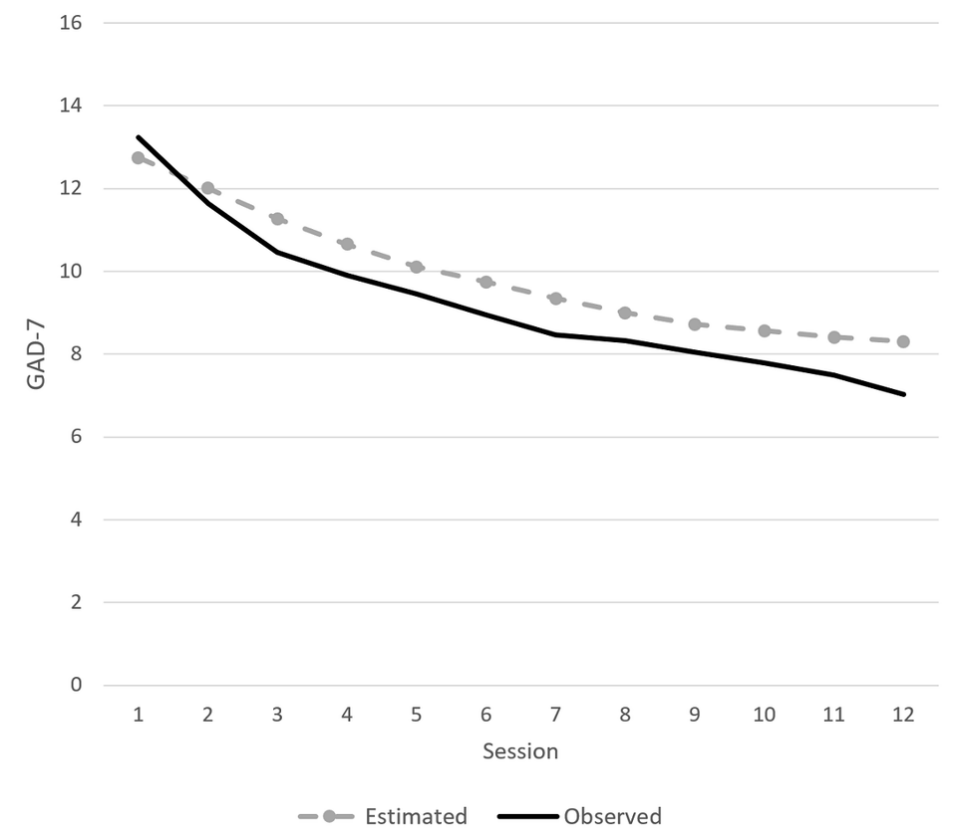
Estimated mean marginal values based on linear mixed model versus observed values. Curves depict an average patient trajectory. GAD-7: Generalized Anxiety Disorder 7-item scale.

The estimated fixed and random effects on GAD-7 are given in Table 2, and the model-estimated mean trajectory is shown in Figure 2. According to the model, after the average time between the pre- and posttreatment observations for completers (171, SD 95 days), the main effects of time on GAD-7 would amount to an average of 3.1 (95% CI 4.4-2.7) points of improvement. The main average effect of the session was 0.523 (95% CI 0.592-0.455) points of improvement per session, or 6.7 (95% CI 5.5-7.1) points after all 12 sessions. Time had a negative interaction with session, and over a complete therapy (12 sessions), an extra week of therapy would amount to an average of 0.20 (95% CI 0.16-0.24) points of deterioration. Older patients improved more than their younger counterparts; for example, 40-year-old patients improved on average 0.3 points more (95% CI 0.1-0.5) than their 20-year-old counterparts after an average course of therapy. Patients from private or occupational health care had similar overall symptoms as patients from student health care (95% CI −0.6 to 1.6) but milder than those from specialized psychiatric care (95% CI 1.0-3.1) and primary health care (95% CI 0.4-1.4). Patients from private and occupational health care benefited similarly as patients from psychiatric services (95% CI −0.12 to 0.17) but more than patients from student (95% CI 0.13-0.46) or primary health care (95% CI 0.05-0.19) per month on therapy. Completers and early dropouts benefited from the treatment similarly (95% CI −0.16 to 0.35), but late dropouts benefited less (95% CI 0.16-0.35) per month. Estimates of random effects indicated that patients likely had individual overall symptom levels (*P*<.001).

**Table 2:** Mixed linear model parameter estimates.

Parameter				Estimate (SE; 95% CI)		Wald Z		*t* test (*df*)		*P* value
**Fixed effects**						N/A^a^				
	Intercept		12.422 (0.210; 12.01 to 12.83)				59.16 (1594.5)		<.001	
	Observation time (time)		−0.028 (0.004; −0.035 to −0.021)				−7.89 (8627.5)		<.001	
	Time^2^		3.35×10^−5^ (1.32×10^−5^; 7.57×10^−6^ to 5.95×10^−5^)				2.53 (8308.9)		.01	
	Time^3^		−3.39×10^−8^ (1.18×10^−8^; −5.70×10^−8^ to −1.07×10^−8^)				−2.87 (8117.6)		.004	
	Observation session (session)		−0.523 (0.035; −0.592 to −0.455)				−15.00 (8652.5)		<.001	
	Time × session		0.002 (0.0003; 0.002 to 0.003)				9.11 (8240.2)		<.001	
	Time × age		−1.32×10^−4^ (4.10×10^−5^; 2×10^−4^ to −5.11×10^−5^)				−3.21 (8669.3)		.001	
	**Referral source, main effect**					N/A				
		Private or occupational	N/A				N/A		N/A	
		Psychiatry	2.059 (0.530; 1.018 to 3.099)				3.88 (1289.4)		<.001	
		Other	1.409 (0.533; 0.363 to 2.454)				2.64 (1272.5)		.008	
		Student health care	0.500 (0.559; −0.597 to 1.598)				0.89 (1336.0)		.37	
		Primary care	0.919 (0.249; 0.430 to 1.408)				3.68 (1303.0)		<.001	
	**Referral source × time**					N/A				
		Private or occupational	N/A				N/A		N/A	
		Psychiatry	0.001 (0.002; −0.004 to 0.006)				0.34 (8163.2)		.73	
		Other	0.004 (0.002; −0.001 to 0.009)				1.58 (8140.2)		.11	
		Student health care	0.010 (0.003; 0.004 to 0.015)				3.46 (8193.8)		.001	
		Primary care	0.004 (0.001; 0.002 to 0.006)				3.51 (8219.9)		<.001	
	**Completion status × time**					N/A				
		Completers, as reference	N/A				N/A		N/A	
		Late dropouts	0.005 (0.001; 0.002 to 0.008)				3.44 (8677.2)		.001	
		Early dropouts	0.003 0.004; −0.005 to 0.012)				0.72 (8525.7)		.47	
**Random effects**										
	Residual		9.40 (0.15; 9.11 to 9.70)		61.76		N/A		<.001	
	Patient intercept		10.56 (0.52; 9.59 to 11.63)		20.34		N/A		<.001	

^a^N/A: not applicable.

### Effect Sizes

Treatment effect sizes are given in Table 3. The estimated ITT treatment effect size was 0.97, indicating a large effect on the GAD-7. The estimated scores indicated a small effect size in early dropouts (Cohen *d*=0.34) and a large effect size in late dropouts (Cohen *d*=0.85). The treatment effect observed for the completers was very large (Cohen *d*=1.39).

At the 3-month follow-up, 111 completers were reached, which accounted for 10.1% of the whole sample and 22.9% of the completers. The observed change in the GAD-7 score was −6.8. For secondary measures, changes were −11.3 in PSWQ and −3.9 in OASIS.

**Table 3:** Treatment effects based on estimated marginal means and observed values.

Treatment effect				Baseline, mean (SD)		Post, mean (SD)		Pre–post, change		Pre–post, correlation		Effect size, Cohen *d* (95% CI)
**GAD-7 ^a^**												
	Completers^b^			13.1 (3.6)		7.0 (4.7)		−6.1		0.267		1.39 (1.25-1.53)
	**Estimated values**											
		Early dropouts	12.9 (3.6)		11.5 (4.5)		−1.3		0.377^c^		0.34 (0.16-0.51)	
		Late dropouts	12.9 (3.6)		9.4 (5.1)		−3.5		0.351^c^		0.85 (0.70-1.00)	
		ITT^d^ analysis	12.9 (3.6)		8.7 (4.7)		−4.1		0.308^c^		0.97 (0.88-1.06)	
**PSWQ^e^**												
	Completers^b^			64.7 (9.9)		53.7 (13.6)		−11.0		0.526		1.14 (1.00-1.27)
**OASIS^f^**												
	Completers^b^			12.0 (2.8)		8.4 (3.9)		−3.6		0.452		1.23 (1.09-1.37)

^a^GAD-7: Generalized Anxiety Disorder 7-item scale.

^b^Fewer patients completed the PSWQ and OASIS after the treatment (n=479).

^c^Only these correlations were calculated from before the treatment and the last observed values for those with ≥2 observations.

^d^ITT: intention to treat.

^e^PSWQ: Penn State Worry Questionnaire. For PSWQ and Overall Anxiety Severity and Impairment Scale, only baseline scores were available for noncompleters; hence, only complete effect sizes were observed.

^f^OASIS: Overall Anxiety Severity and Impairment Scale.

### Clinical Change Indexes

Clinical change indexes based on the GAD-7 scores are provided in Table 4. After the treatment, 23.29% (256/1099) of patients achieved remission (GAD-7 score <5).

Indexes for PSWQ and OASIS were reported only for completers because noncompleters had only one observation. Reliable change on PSWQ was a change of >12 points and 40.9% (196/479) reliably improved and 1% (5/479) reliably deteriorated. On OASIS reliable change was a change of >3 points and 48.6% (233/479) reliably improved and 1.5% (7/479) reliably deteriorated.

**Table 4:** Clinical indexes at the final on-therapy observation.

Clinical index			ITT^a^, n (%)		Completers, n (%)		Late dropouts^b^ (sessions ≥4), n (%)		Early dropouts^b^ (sessions <4), n (%)
**Baseline GAD^c^** **-7 score ≥8**			1099 (100)		485 (100)		363 (100)		251 (100)
	Reliable improvement^d^	532 (48.4)		306 (63.1)		170 (46.8)		56 (22.3)	
	Reliable deterioration^e^	41 (3.73)		12 (2.5)		19 (5.2)		10 (4)	
**Baseline GAD-7 score ≥10**			894 (100)		385 (100)		295 (100)		214 (100)
	Reliable improvement^d^	479 (53.6)		268 (69.6)		155 (52.5)		56 (26.2)	
	Recovery^f^	486 (54.4)		280 (72.7)		149 (50.5)		57 (26.6)	
	Reliable recovery^g^	421 (47.1)		244 (63.4)		129 (43.7)		48 (22.4)	
	Reliable deterioration^e^	27 (3)		9 (2.3)		11 (3.7)		7 (3.3)	

^a^ITT: intention to treat.

^b^On the basis of the last observed values.

^c^GAD-7: Generalized Anxiety Disorder 7-item scale.

^d^GAD-7 score drop of ≥5.

^e^GAD-7 score increase of ≥5.

^f^GAD-7 changed to <10 at after the treatment.

^g^Both reliably improved and recovered.

## Discussion

### Principal Findings

This nationwide, free-of-charge, therapist-supported HUS-iCBT for GAD in routine care, with no predetermined maximum completion time, comprised 1099 patients referred by their physicians. To the best of our knowledge, this study has the largest reported real-world sample involving iCBT for GAD. The patients reported substantial improvement in ITT analyses amounting to effect sizes of Cohen *d*=0.97 on GAD-7; and for completers, Cohen *d*=1.39 on GAD-7, Cohen *d*=1.14 on PSWQ, and Cohen *d*=1.23 on OASIS. The full completion rate was 44.1% (485/1099), and the average session completion rate was 7.8 (65%) of the total 12 sessions. A reliable improvement occurred in 48.4% (532/1099) of the full sample. Of those with a baseline GAD-7 score ≥10, reliable improvement occurred in 53.6% (479/894), with a transition to recovery achieved by 54.4% (421/894). These results seemed to be enduring for 22% (111/485) of completers who answered the follow-up questionnaires. The average change on the GAD-7 at 3-month follow-up was still −6.8 points (vs −6.1 at after the treatment).

### Comparison With Earlier Work

#### Symptomatic Change

The change in GAD-7 score was comparable with that achieved in 3 of the 4 Australian ThisWayUp trials (Cohen *d*=0.91-1.18 [23,26,27]) lower than that in the fourth trial that used different inclusion criteria (Hedges *g*=2.06 and 2.10 [28]), and comparable with the Online Therapy Unit’s trial (Cohen *d*=1.07 [29]). The change in GAD-7 scores in our study was also not inferior to the within-group change in a recent meta-analysis of RCTs of transdiagnostic iCBT for anxiety and depression (95% CI 0.91-1.22 [15]). The effect size also seems encouraging when contrasted with change in comparison with a waiting list in another meta-analysis (95% CI 0.39-1.01 [9]).

Interestingly, from both practical and theoretical viewpoints, the longer it took for patients to complete a given number of sessions, the less they improved. It is plausible that the effects of therapy become diluted if the patient’s commitment to therapy weakens. Therefore, methods of increasing engagement in therapy seem one of the most promising ways to increase the effectiveness of iCBT for GAD.

The change in the PSWQ for completers (Cohen *d*=1.14) indicates a substantial improvement in worry. In a meta-analysis of 10 RCTs of iCBT for GAD, the ITT effect size on the PSWQ in iCBT was 0.71 [16]. Although the comparison is not direct, the results of this study agree with the existing RCT results.

The OASIS-measured anxiety-related functional impairment demonstrated a large improvement in the completers (Cohen *d*=1.23). Of the ThisWayUp trials, Mahoney et al [28] found medium to large improvements in health-related disability and functioning, as measured by the World Health Organization Disability Assessment Schedule 2.0. Similarly, a trial conducted by the Online Therapy Unit [29] found that patients had a large decrease in functional impairment after therapy, as measured by the Work and Social Adjustment Scale. The combined results strongly suggest that iCBT for GAD can improve functional capacity.

Our completers improved faster than late dropouts, unlike those in 2 earlier studies, which showed no or negligible differences. However, significant improvement was also observed in our noncompleters, which could imply heterogeneity in this population. Feedback offered by the patients who withdrew from the Online Therapy Unit intervention also suggests that several patients may have discontinued treatment as their symptoms improved [29]. In iCBT for depression, there seems to be a subgroup of patients who benefit from the therapy rapidly and discontinue treatment prematurely [56]. These patients may regard further treatment as unnecessary. Hence, discontinuation should not be considered, by default, a failure.

Although not unequivocally proven, clinician-referred patients in iCBT tend to exhibit lower effect sizes than community-recruited or self-referred ones [13,57]. Thus, our results seem encouraging, as all patients in HUS-iCBT have been physician-referred.

#### Recovery and Reliable Change

In the ITT analysis using LOCF, of HUS-iCBT patients with a baseline GAD-7 score ≥10, 54.4% (486/894) of patients achieved recovery. This recovery rate is comparable with that reported earlier in face-to-face CBT for GAD (51.4% [54]). Owing to differing imputation methods, we cannot directly compare our ITT results with those of Australian studies. Nevertheless, Hobbs et al [26] reported a 70% recovery rate for completers, which is comparable with our 72.7% (280/385).

Moreover, the criteria for reliable change in our study and previous studies differed, which prevented direct comparison. In one meta-analysis of RCTs of iCBT, reliable deterioration occurred in 3.1% (95% CI 1.5%-5.9%) of patients in trials for anxiety disorders [13]; in another meta-analysis of several disorders, reliable deterioration occurred in 5.8% of iCBT patients (vs 17.4% of patients on the waiting list) [21]. In HUS-iCBT, reliable deterioration occurred in 3.73% (41/1099) of patients. Thus, HUS-iCBT for GAD also appeared to be as safe as precious treatments and safer than no treatment.

#### Adherence

The full completion rate in our study (485/1099, 44.13%) was similar to the rates in previous routine care trials (36%-55%) but lower than that in a meta-analysis of routine care iCBT for depression and anxiety (61%) [13]. However, RCTs are likely to inflate adherence when compared with real-world trials. For instance, ThisWayUp’s trials have shown a dramatic decrease from very high full completion rates when transitioning from a research setting into primary care (75%-85% vs 38% [24]).

The proportion of completed sessions in HUS-iCBT (65%) was marginally lower than that in the earlier trials (67%-77% in ThisWayUp’s and 72% in Online Therapy Unit’s trials) but somewhat higher than that in the previously mentioned meta-analysis (57% [13]). Compared with ThisWayUp’s program, a lower proportion of completed sessions may be expected in a therapy comprising twice as many sessions (12 vs 6). Interestingly, more patients in the HUS-iCBT completed ≥6 sessions compared with the Australian trials (61% vs 36%-55%). On the other hand, the Online Therapy Unit had slightly better results with a program of similar length both in sessions (n=12) and average days on therapy (135 vs our 128). Differences in the course of therapy, recruitment, and therapist protocols prevent straightforward comparison. The comparison is further hampered by the overlapping recruiting periods in the Australian trials, as the number of patients reported remains unique. Hence, the optimal number of iCBT sessions for GAD remains unknown.

Compared with our noncompletion rate (614/1099, 55.86%), face-to-face individual psychotherapy for GAD has had a low dropout rate of 17% in a meta-analysis of RCT trials [58], and a meta-analysis of nonrandomized outpatient studies on CBT for adult anxiety disorders showed a similar dropout rate of 15% [59]. However, the criteria for completion in face-to-face psychotherapy are often more lenient, such as stopping when agreed upon with the therapist or completing a minimum number of sessions [59]. Thus, the adherence numbers are not comparable and are likely to be closer to iCBTs when analyzed with an equivalent metric.

In our study, completers sent and received more messages than did noncompleters. Our data do not allow exploration of causality, but this finding may be intuitively explained by the completer’s longer time on therapy. In the Online Therapy Unit’s trial [29], both patients and therapists sent more messages (on average 9.6 and 20.0, respectively) than ours (4.2 and 8.4, respectively). This difference is not surprising, given the differences in intake protocol (both clinician referral and community recruitment and intake interview vs strictly a physician’s referral and only referral screening in HUS-iCBT) and therapist-contact intensity (weekly messages vs minimum 4 messages per therapy). These differences may also partly explain the higher adherence in the Online Therapy Unit’s trial.

#### Patients’ Background Effects

Older patients improved more and faster than younger patients. At first glance, this seems counterintuitive, as one could presume that young, digital native patients could feel more comfortable in the digital realm than the older ones. Nevertheless, 1 of the 3 ThisWayUp’s trials also found that older age predicted larger improvement [23], whereas another found no such relationship [26], and the third trial found a relationship but left its direction unreported [27]. Furthermore, in a transdiagnostic study of iCBT for anxiety and depression, older age predicted greater improvement [60], whereas in a meta-analysis of various psychotherapies for GAD, older participants improved less than younger ones [61].

Moreover, younger patients were less likely to complete the program than their older counterparts. This relationship has been a common finding in iCBT for GAD in routine care [23,26,27] but not in face-to-face psychotherapy for GAD [58]. The average age in this study was 33 years, 6 or 7 years lower than that in the 3 ThisWayUp and single Online Therapy Unit study that reported their sample ages [23,26,27,29]. Overall, research on the age issue is scarce, and what still remains obscure is how age may or may not affect the results of different modes of therapy, such as face-to-face therapy or iCBT.

Gender had no significant influence on adherence in our study, similar to the findings of Australian studies. Systematic reviews of RCTs on face-to-face psychotherapy for GAD or CBT for anxiety disorders have also found no significant gender-adherence relationship [54,58].

The full completion rate was higher among patients referred from private or occupational health (171/339, 50.4%) than among those referred from primary care (235/601, 39.1%). Their symptoms improved faster than those referred from students or primary health care. These effects could be due to patient-group differences: patients referred from occupational health are, by definition, employed and presumably have a higher average level of functioning, higher socioeconomic status, and a lower likelihood of serious comorbidities. Moreover, in our recent study, physicians in occupational health displayed more interest in sending patients to iCBT than their primary care counterparts [62], which may transfer into patients’ own expectations and motivation.

Patients from nonurban municipalities were more likely to complete the therapy (73/130, 56.2%) than those from urban municipalities (412/966, 42.7%). This relationship is somewhat opposed to the findings in the 2012 report by Mewton et al [23], where patients from urban settings were more likely to complete the therapy than were those from a rural setting. The causes of this difference are unknown and are likely to include multiple recently discovered factors, such as differences in technology adoption, financial concerns, and access to treatment [63]. Nevertheless, the municipality class did not demonstrate a significant impact on symptomatic improvement in the mixed model analysis.

#### Design Comparison

The results of this and earlier studies in routine care are, in general, comparable despite considerable differences in setting, design, and support.

To the best of our knowledge, HUS-iCBT and the Online Therapy Unit’s programs had no predetermined maximum therapy completion time, whereas ThisWayUp applied a typical fixed maximum time restriction (90 days). The time from the first to last observation on HUS-iCBT (128 days) and from the first to last log-in at the Online Therapy Unit (135 days) were comparable and longer than the typical 90 days in other trials. Although the lack of a predetermined maximum time span may support adherence during changing life situations, it could also disengage some patients and dilute therapy effects, thereby leading to an increased dropout rate. As confounding design and sample features exist, such as different numbers of sessions and differences in sample average age, the optimal time span remains unknown.

In the Australian studies, patients were referred to iCBT by their own independent clinicians, each of whom was required to register as a provider, to receive training, and to use an assessment toolkit. The Online Therapy Unit required a centralized diagnostic interview. Our HUS-iCBT only screened referrals from physicians (but not from other clinicians) with no obligatory registration or any specific assessment schema. The practice at HUS-iCBT was chosen as the middle ground to ensure proper diagnostics while maintaining a high intake flow. To maintain a low threshold, the inclusion criteria in HUS-iCBT were purposely loose, with only 0.6% (51/8394) of all physician referrals rejected during the recruitment period, and this may have contributed to a possible patient–therapy mismatch.

In HUS-iCBT, the support was provided centrally by specially trained and supervised mental health professionals, as recommended by authors from 5 universities who argue that iCBT requires specialized expertise and processes [64]. In the ThisWayUp studies, support was provided by the referring clinicians, who rarely contacted their patients. A pre-existing alliance could compensate for the clinicians’ lack of specialized training.

HUS-iCBT was free of charge for the patients, as was the treatment at the Online Therapy Unit. Patients in ThisWayUp paid approximately Aus $49 (US $37) for the treatment, which may have ensured motivation and therefore improved adherence [24] but may also serve as a barrier to treatment.

### Strengths and Limitations

The main strengths of this study are its nationwide scope, the largest sample thus far reported, and its routine care setting. One could argue that reliance on self-report measures can inflate treatment effects, but 2 recent meta-analyses suggest that combining indexes based on self-reporting can be even conservatively biased [7,54]. Self-report measures also fulfill practical requirements for scalability.

Other routine care studies did not use PSWQ or OASIS. Their addition in our study shows that iCBT for GAD can ease pathological worry and anxiety-related impairments in routine care. Although they both offer important information, they may not be as sensitive to change as the GAD-7.

Concomitant treatments, including psychopharmaceuticals, are often offered to patients in routine care. Information on these treatments is not reliable. This limits our understanding of the possible interactions between different treatments.

Not all comorbidities may have appeared in patient documents in referrals, and depressive symptoms were not measured. This issue is particularly relevant for GAD, as its annual comorbidity with major depression is around 41% [4]. One effectiveness study also indicated that depressive symptoms could decrease treatment effectiveness [26]. The lack of depression measurement does not weaken the study’s results but highlights the question of whether every one of our patients received optimal treatment.

We could not, retrospectively, reliably identify the therapist behind each message sent to the patient. We know that due to vacation periods, illness, or leaving HUS, not all messages sent to a patient may have come from the same therapist. This prevented us from analyzing the effect of the individual therapist, their profession, or other relevant training.

Only 23% (111/485) of completers were reached for the 3-month follow-up. Such high attrition limits our conclusions regarding long-term effectiveness. However, we did not include follow-up data in the linear mixed model analyses, and these limited data do not compromise the main results.

### Future Research

Thus far, therapist-supported iCBT for GAD has not been compared with other active treatment forms or no treatment in routine care, making comparisons with active controls, coupled with health economic analyses, essential. Not all patients benefit from iCBT for GAD. Both transdiagnostic programs and tailoring content to individual needs have displayed promising results [15,64], although for anxiety, unguided transdiagnostic iCBTs may be less or no more efficacious than diagnosis-specific ones [19,35]. Tailored transdiagnostic treatments seem likely to be a significant trend in iCBT, but evidence for their added value still needs to be confirmed.

Adherence to iCBT for GAD seems to be lower in primary care than in RCT trials. Evidence is emerging for adapted therapist support for patients at risk for dropout [65,66] and for an optimal therapist-contact schedule for iCBT in cases of depression and insomnia [67,68]. Machine learning applications could accelerate the identification of patients at risk for dropout and accelerate proactive support. However, this venue of research is still in its infancy.

iCBT programs often retain a considerable number of text-based answers and messages. Text mining approaches could offer an exciting avenue for exploring qualitative phenomena. Such analyses could be beneficial for both theoretical research and the practical application of iCBT.

The optimal time schedules for or the number of sessions in iCBT for GAD remain unclear. Further comparison studies should seek to establish more suitable, effective, and economic combinations. Age and other demographic variables may also influence the outcomes of iCBT for GAD. To provide equally effective care for everyone, future studies should investigate how to overcome hurdles presented by demographic variables.

PSWQ and OASIS were not used in other routine care studies. They represent both diagnosis-specific and transdiagnostic processes related to GAD and anxiety in general. OASIS, a short self-rating scale, opens up a venue for further comparative research between iCBT for various psychiatric disorders. As PSWQ measures pathological worry, a theoretically important GAD feature, an in-depth psychometric examination of PSWQ may reveal important information on moderators and mediators of effectiveness.

Since the collection of our data, HUS-iCBT for GAD has adopted a novel technical platform, a predetermined maximum completion time (20 weeks), pretreatment phone calls, and weekly therapist support. These changes may have practical implications that will enable further studies.

### Conclusions

This nationwide, free-of-charge, therapist-supported HUS-iCBT for GAD with no predetermined maximum completion time was effective at improving symptoms and reducing worry and functional impairment in routine care. Overall, this therapy appears to be safer than no treatment and is at least as effective as other therapist-supported iCBTs for GAD. The observed gains at the 3-month follow-up should be confirmed in future studies. Future research needs to establish comparative effectiveness against other treatments and to optimize the benefit of iCBT for GAD in a variety of patient groups and individual patients.

## Appendix

Multimedia Appendix 1 Description of primary mixed model building and statistics.

## Abbreviations

CBT: cognitive behavioral therapy
GAD: generalized anxiety disorder
GAD-7: Generalized Anxiety Disorder 7-item scale
iCBT: internet-delivered cognitive behavioral therapy
ITT: intention to treat
LOCF: last observation carried forward
OASIS: Overall Anxiety Severity and Impairment Scale
PSWQ: Penn State Worry Questionnaire
RCI: Reliable Change Index
RCT: randomized controlled trial
