# Supplementary material for: Internet-Delivered Cognitive Behavioral Therapy for Generalized Anxiety Disorder in Nationwide Routine Care: Effectiveness Study
Source: J Med Internet Res. 2022 Mar 24;24(3):e29384. doi: 10.2196/29384 (PMC8990365; doi:10.2196/29384)
Supplement: Multimedia Appendix 1 [file jmir_v24i3e29384_app1.docx]

Appendix 1

The final model was built backward stepwise. First, we tested full models 1 and 2 and decided to keep session number as a predictor in addition to observation time. Afterwards we pruned the model by excluding factors under *P*<.01 significance, while making sure that both AIC and BIC don’t show worsening fit. Δ

| ***Table 1.*** *Primary mixed linear model testing* |  |  |  |
| --- | --- | --- | --- |
| Model | -2 log likelihood | AIC | BIC |
| Intercept | 48565,7186 | 48571,7186 | 48592,9249 |
| + observation time | Δ -1805,7186 | Δ -1799,2766 | Δ -1778,0702 |
|  |  |  |  |
| Full Model 1 (observation time, age, gender, referral source, municipality class, completion status + interactions with observation time) | Δ -145,0039 | - Δ 105,4459 | Δ 35,9295 |
| Full Model 2 (+observation session and interactions with observation time and completion status) | Δ -214,9294 | Δ -206,9294 | Δ -178,6543 |
|  |  |  |  |
| Model 2.1 (- Gender x Observation time) | Δ 0,0005 | Δ -1,9994 | Δ -9,0682 |
| Model 2.2 (- Municipality class) | Δ 0,7228 | Δ -3,2771 | Δ -17,4147 |
| Model 2.3 (- Municipality class x observation time) | Δ 1,1639 | Δ -2,8355 | Δ -16,973 |
| Model 2.4 (- Age) | Δ 2,1960 | Δ 0,1955 | Δ -6,8732 |
| Model 2.5 (- Gender) | Δ 2,6630 | Δ 0,6630 | Δ -6,4057 |
| Model 2.6 (- Completion Status x Observation session) | Δ 5,2324 | Δ 1,2324 | Δ -12,9051 |
| **Model 2.7 (- Completion Status)** | Δ 7,1171 | Δ 3,1171 | Δ -11,0204 |
|  |  |  |  |
